# Supplementary material for: Structure, mechanism and crystallographic fragment screening of the SARS-CoV-2 NSP13 helicase
Source: Nat Commun. 2021 Aug 11;12:4848. doi: 10.1038/s41467-021-25166-6 (PMC8358061; doi:10.1038/s41467-021-25166-6)
Supplement: Supplementary file 1 — Supplementary information [file 41467_2021_25166_MOESM1_ESM.pdf]

Supplementary Information: Structure, Mechanism and Crystallographic fragment screening of the SARS-CoV-2 NSP13 helicase

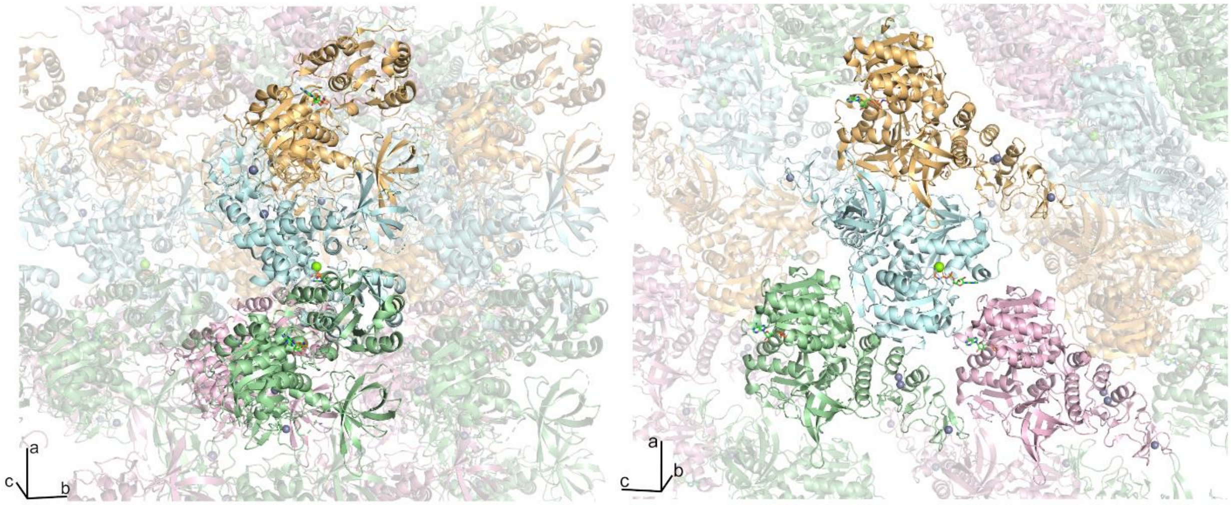

Supplementary Figure 1 – Overview of the crystal packing interactions for the 4 chains in the asymmetric unit of the AMP-PNP crystals viewed from two perpendicular angles. Chains A, B, C and D have been coloured Cyan, Orange, Green and Pink respectively.

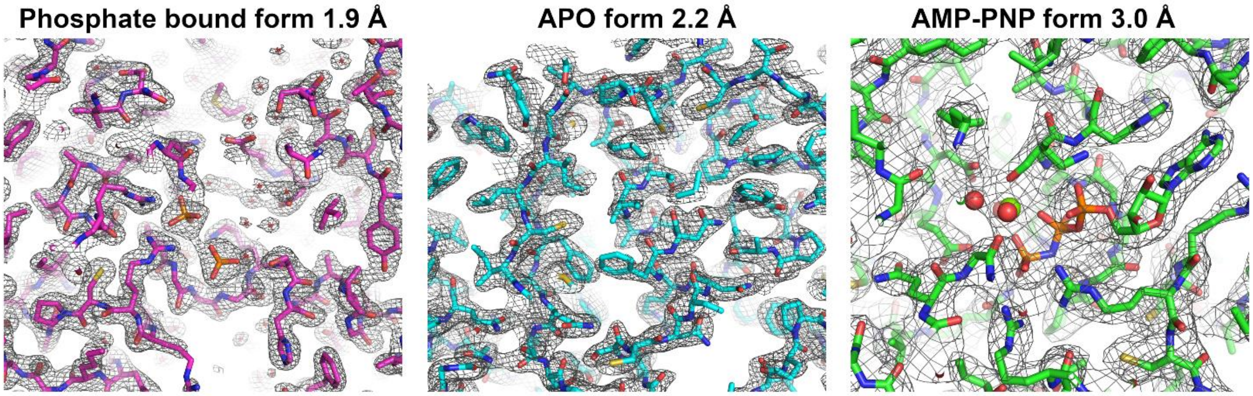

Supplementary Figure 2 – Representative $2F_o$-$1F_c$ electron density maps of the various NSP13 crystals contoured at 1.2 σ.

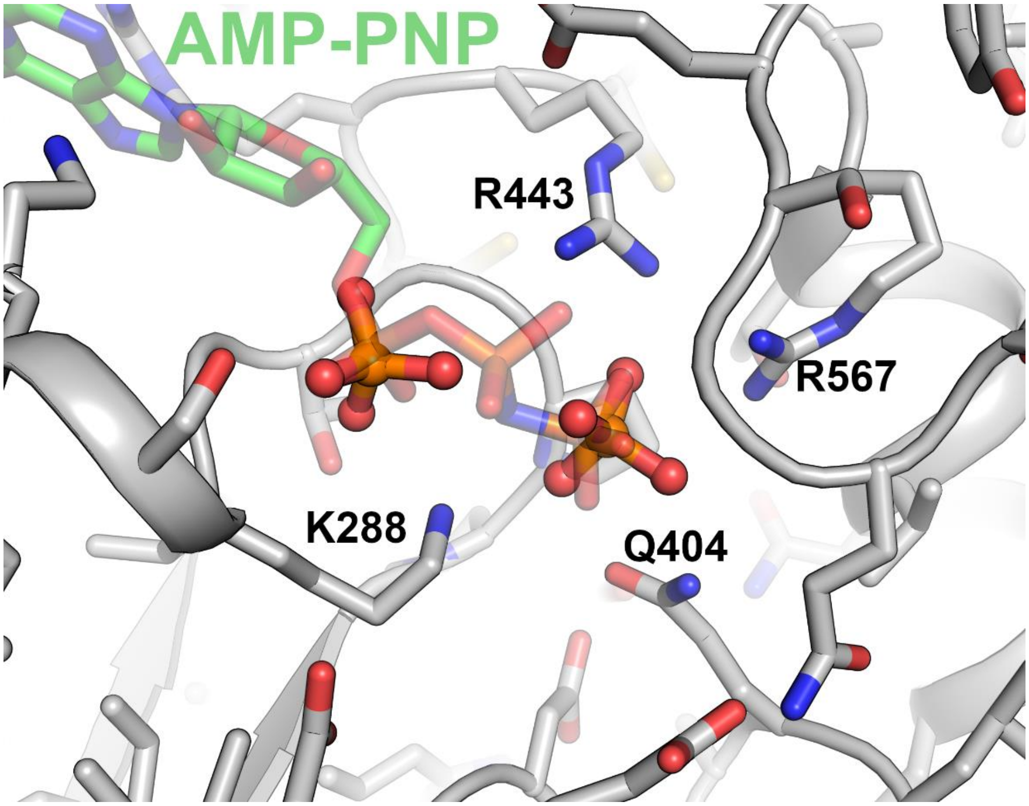

Supplementary Figure 3 – Comparison of the position of the phosphate ions in the phosphate bound crystals with the AMP-PNP in binding mode B. The AMP-PNP is shown in semi-transparent green with the two phosphates in the phosphate bound form occupying positions equivalent to the σ and γ.

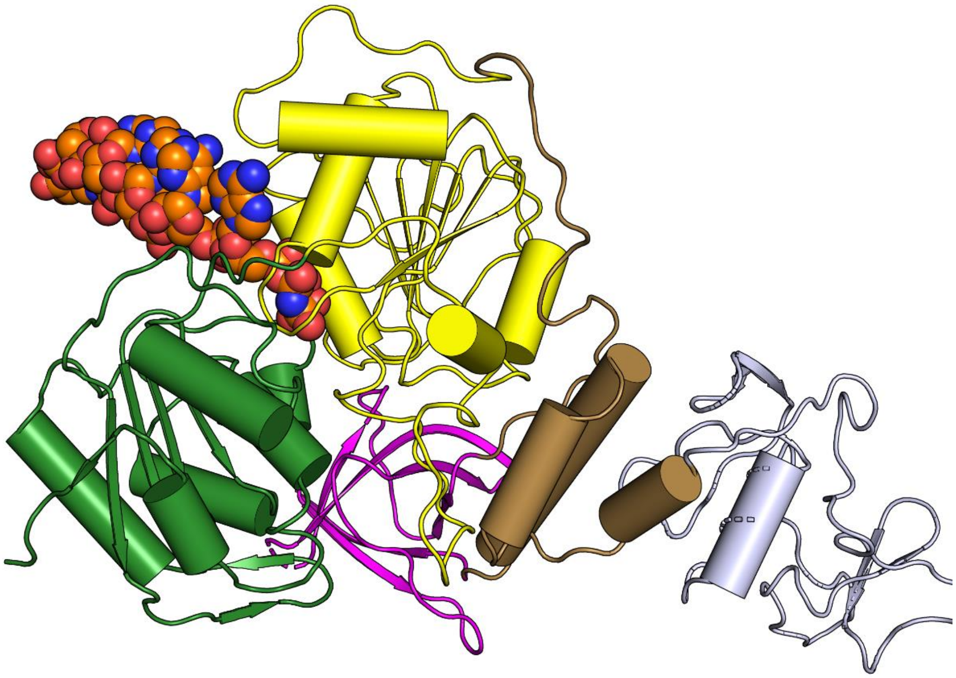

Supplementary Figure 4 – Model of a NSP13 accommodating an RNA containing a 5' triphosphate into the NSP13 ATPase active site. The model was constructed by connecting a short RNA template with the free 3' OH of the AMP-PNP nucleotide which points toward the solvent in binding mode A and can be connected without any severe steric clashes.

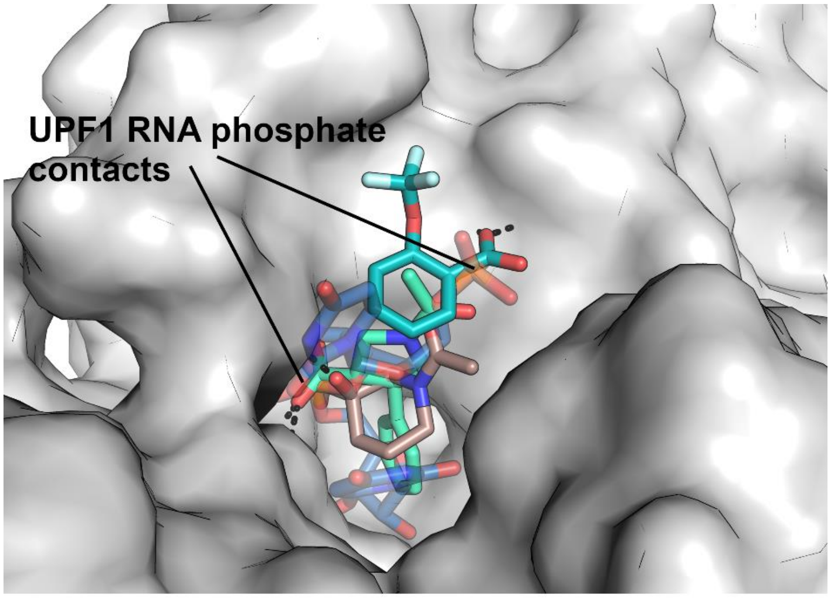

Supplementary Figure 5 – Fragments in the 5' end of the RNA binding channel make contacts to the protein that are shared by conserved RNA phosphate interactions in the UPF-1 RNA structure. The RNA is shown in semi-transparent blue with phosphate positions indicated.

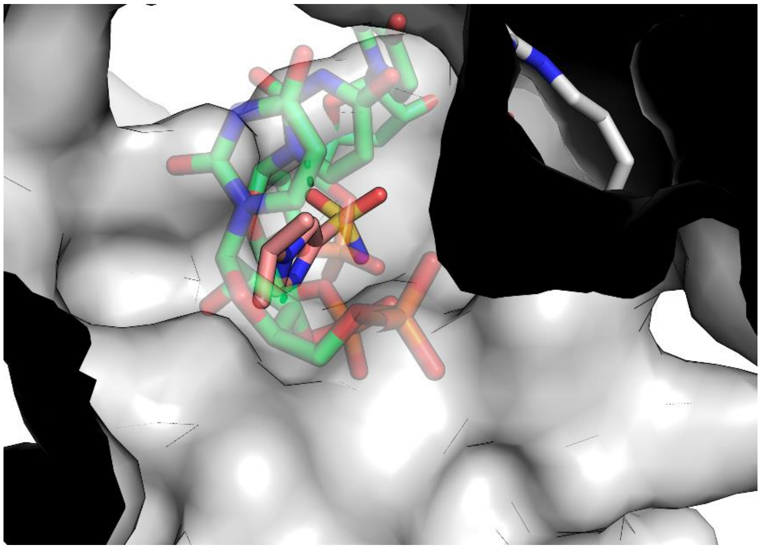

Supplementary Figure 6 – A Fragment in the central RNA binding channel makes contacts to the protein that are shared by conserved RNA phosphate interactions in the UPF-1 RNA structure. The RNA is shown in semi-transparent green.

	This study								SARS–CoV-1		Cryo-EM structures				
	Phos-A	Phos-B	APOA-A	APO-B	PNP-A	PNP-B	PNP-C	PNP-D	6JYT-A	6JYT-B	6XEZ-1	6XEZ-2	7CXM-1	7CXM-2	7CYQ-1
Phos-A	0	1	1.227	0.569	1.843	0.966	2.11	1.26	1.637	1.759	2.57	2.75	2.611	2.752	1.789
Phos-B		0	0.522	1.264	2.243	0.925	1.24	0.663	1.606	1.667	2.037	2.861	2.104	2.839	1.314
APO-A			0	1.331	2.406	1.02	1.05	0.913	1.654	1.638	2.287	2.805	2.335	2.871	1.562
APO-B				0	1.58	0.871	1.888	1.318	1.104	1.226	2.403	2.609	2.408	2.553	1.911
PNP-A					0	2.17	3.05	2.41	2.578	2.575	3.112	2.072	3.002	1.649	2.435
PNP-B						0	1.25	0.67	1.118	1.246	2.028	2.986	2.135	3.019	1.895
PNP-C							0	0.75	1.5	1.536	2.006	3.327	2.141	3.46	1.92
PNP-D								0	1.141	1.189	1.857	3.007	1.996	3.083	1.566
6JYT-A									0	0.382	1.838	3.334	1.969	3.399	2.156
6JYT-B										0	1.717	3.077	1.901	3.232	2.156
6XEZ-1											0	2.849	0.866	3.227	2.24
6XEZ-2												0	2.744	0.843	2.768
7CXM-1													0	2.878	2.18
7CXM-2														0	2.742
7CYQ-1															0

Supplementary Table 1 – Pairwise RMSD values for overall structure comparisons of individual NSP13 chains. Chains from this study are references by chain ID for the phosphate bound (Phos), APO (APO) and AMP-PNP (PNP) bound forms whilst other structures are referenced by their PDB ID.

PDBID	Ligand	Binding Location	Binding Pocket	Resolution (Å)
5RL7	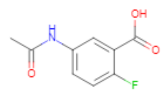 Z364321922	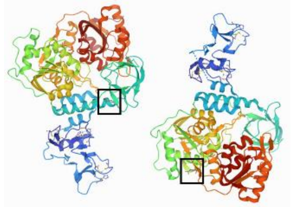 Nucleotide pocket A & RNA 3' B	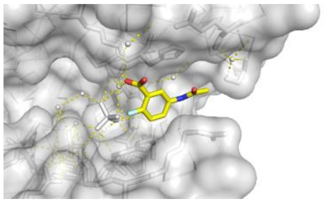 Nucleotide pocket A	1.89
5RLV	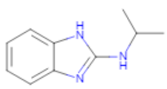 Z2467208649	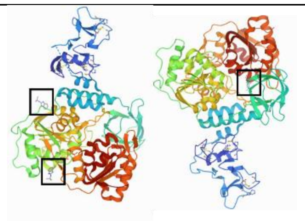 Nucleotide pocket A & Other A & RNA 5' proximal B	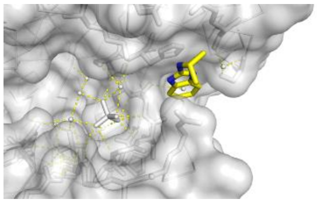 Nucleotide pocket A	2.21
5RLY	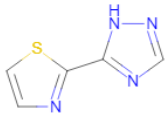 Z2027049478	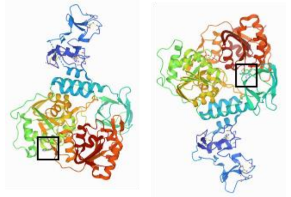 Nucleotide pocket A & RNA 5' proximal B	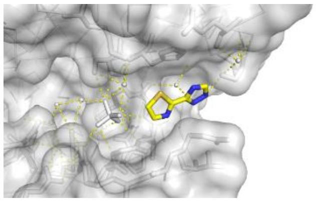 Nucleotide pocket A	2.43

5RLS	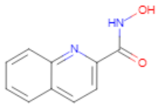 Z59181945	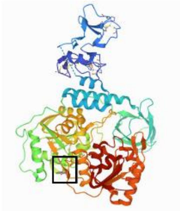 Nucleotide pocket A	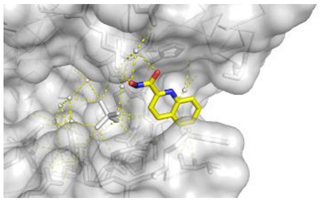 Nucleotide pocket A	2.28
5RLN	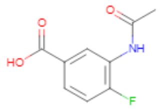 Z364328788	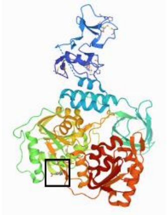 Nucleotide pocket A	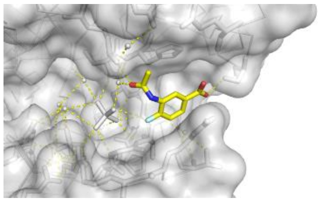 Nucleotide pocket A	2.15
7NNG	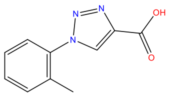 Z2327226104	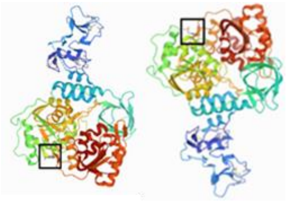 Nucleotide pocket A & B	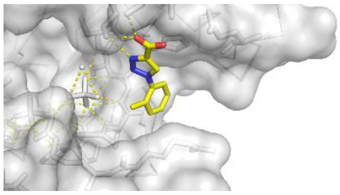 Nucleotide pocket A	2.38
5RL9	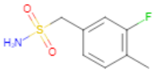 Z1703168683	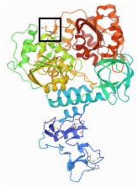 Nucleotide pocket B	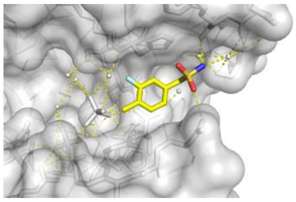 Nucleotide pocket B	1.79
5RLI	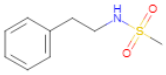 Z45617795	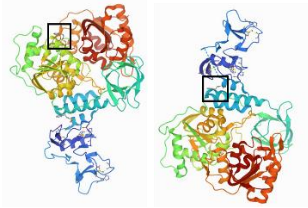 Nucleotide pocket B & Stalk A	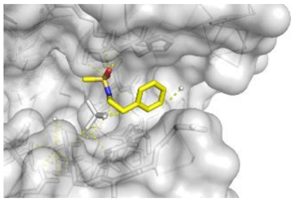 Nucleotide pocket B	2.26
5RLJ	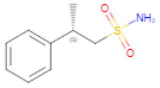 Z1407673036	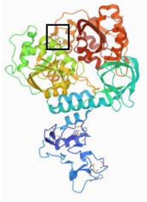 Nucleotide pocket B	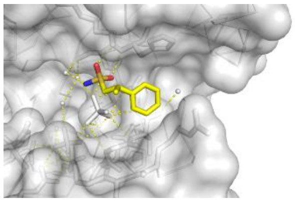 Nucleotide pocket B	1.88

5RLO	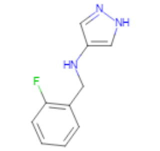 Z1454310449	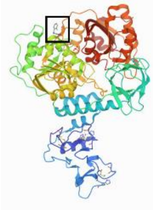 Nucleotide pocket B	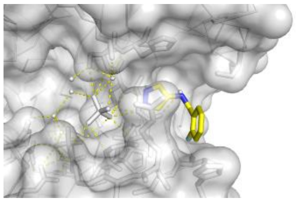 Nucleotide pocket B	2.10
5RLR	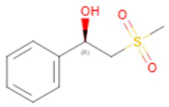 Z822382694	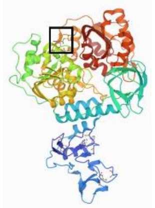 Nucleotide pocket B	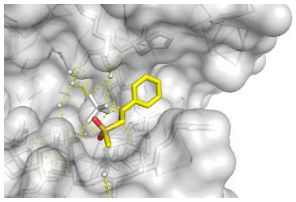 Nucleotide pocket B	2.32
5RLW	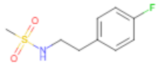 Z45705015	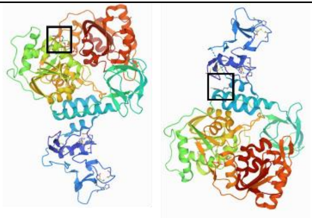 Nucleotide pocket B & Stalk A	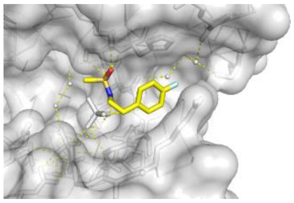 Nucleotide pocket B	1.97
5RM2	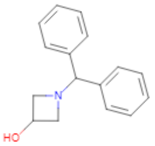 Z1741964527	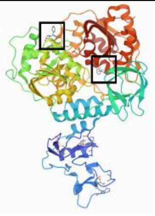 Nucleotide pocket B & RNA 5' Proximal B	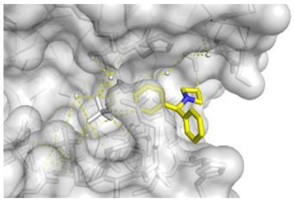 Nucleotide pocket B	1.82
5RM7	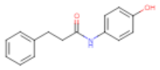 Z69118333	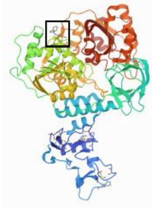 Nucleotide pocket B	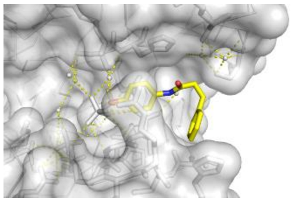 Nucleotide pocket B	1.84
5RLL	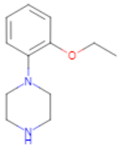 Z425387594	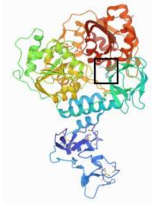 RNA 5' Proximal B	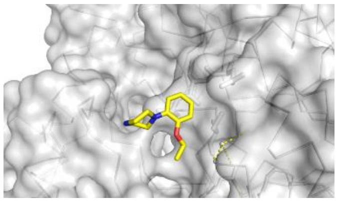 RNA 5' Proximal B	2.08

5RLE	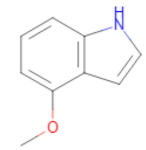<br>Z1429867185	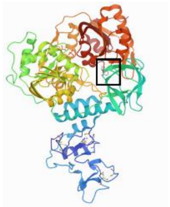<br>RNA 5’ Proximal B	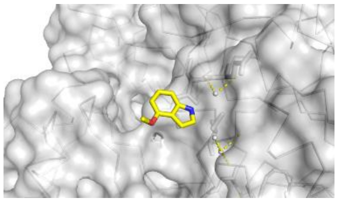<br>RNA 5’ Proximal B	2.27
5RLP	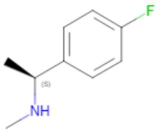<br>Z166605480	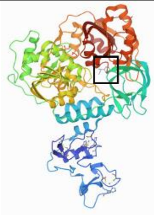<br>RNA 5’ Proximal B	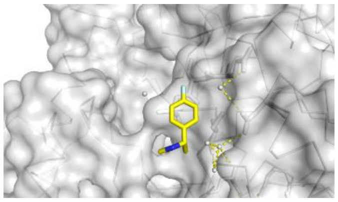<br>RNA 5’ Proximal B	2.56
5RMK	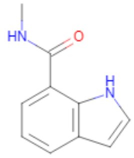<br>Z1273312153	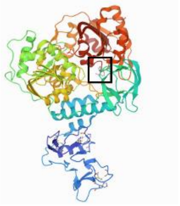<br>RNA 5’ Proximal B	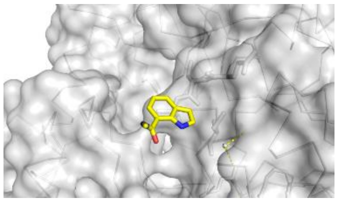<br>RNA 5’ Proximal B	2.08
5RLH	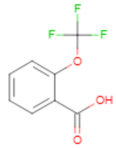<br>Z2856434778	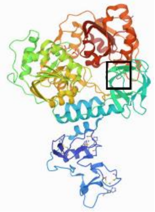<br>RNA 5’ B	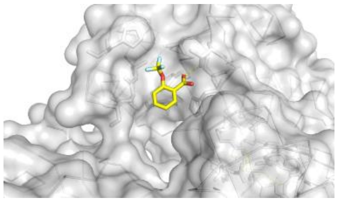<br>RNA 5’ B	2.38
5RMM	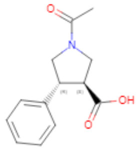<br>POB0066	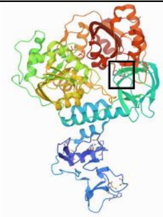<br>RNA 5’ B	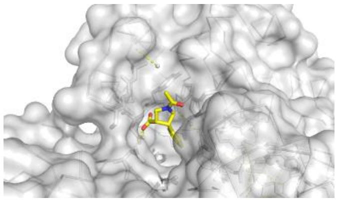<br>RNA 5’ B	2.20
5RLZ	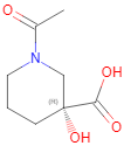<br>Z2293643386	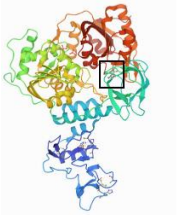<br>RNA 5’ B	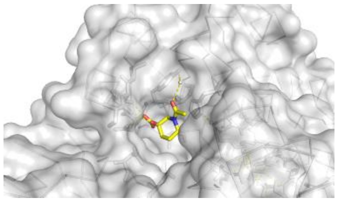<br>RNA 5’ B	1.97

5RL6	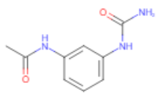 Z198195770	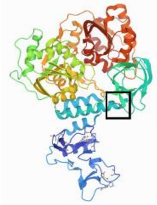 RNA 3’ B	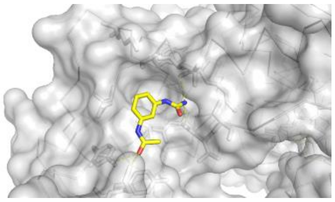 RNA 3’ B	1.92
5RLU	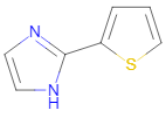 Z744754722	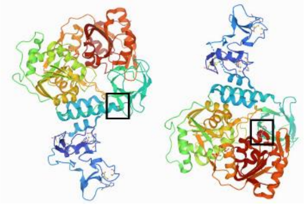 RNA 3’ B & Other A	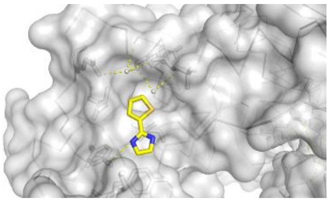 RNA 3’ B	2.35
5RL8	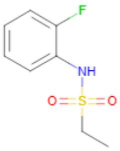 Z53825177	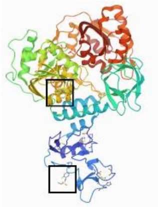 RNA 3’ B & Zinc B	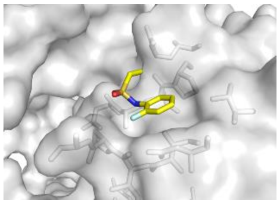 RNA 3’ B	2.21
5RMC	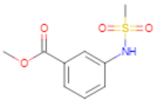 Z24758179	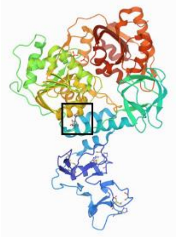 RNA 3’ B	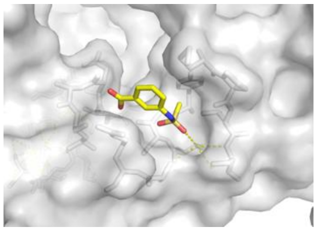 RNA 3’ B	2.15
5RLK	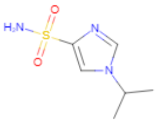 Z1509882419	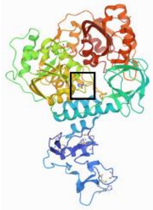 RNA central B	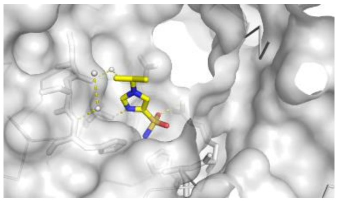 RNA central B	1.96
5RML	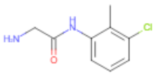 Z85956652	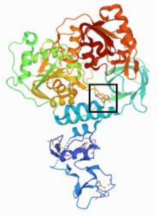 RNA central A	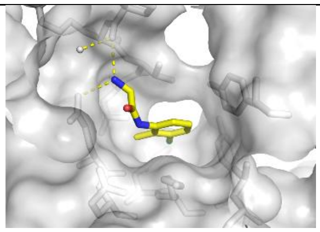 RNA central A	2.43

5RLB	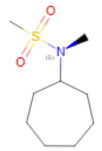 Z216450634	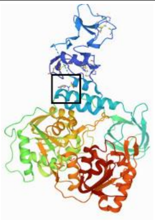 Stalk A	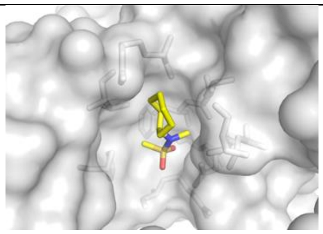 Stalk A	1.98
5RMD	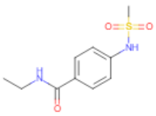 Z57614330	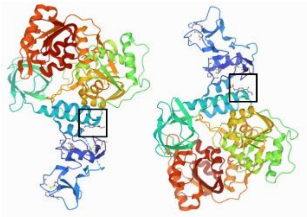 Stalk A & Stalk B	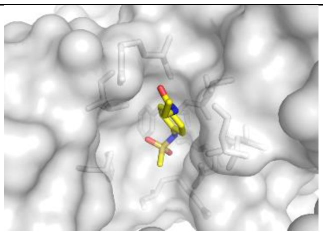 Stalk A	1.92
5RLC	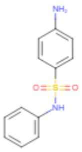 Z56923284	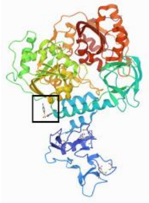 Stalk B	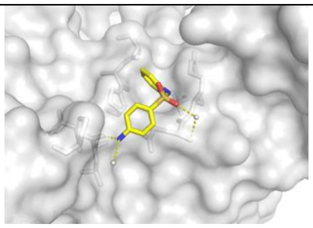 Stalk B	1.92
5RLD	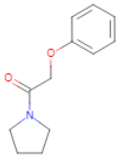 Z19735981	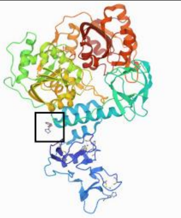 Stalk B	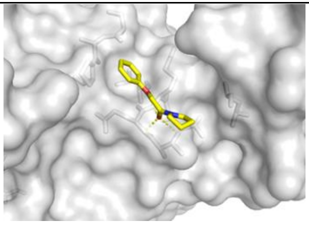 Stalk B	2.23
5RM0	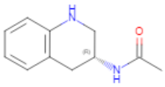 Z1492796719	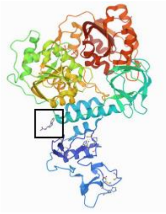 Stalk B	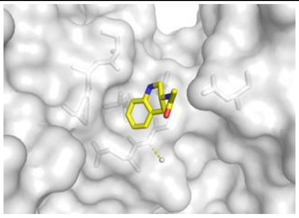 Stalk B	1.91
5RM1	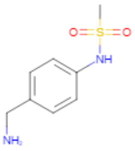 Z426041412	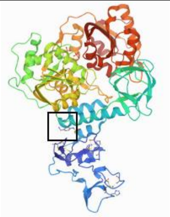 Stalk B	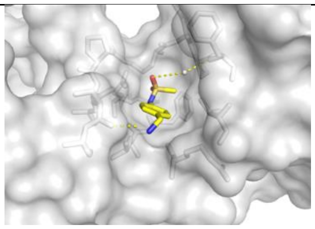 Stalk B	1.90

5RME	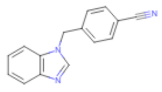<br>Z26333434	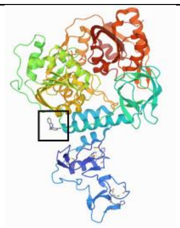<br>Stalk B	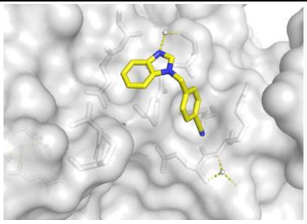<br>Stalk B	2.23
5RLT	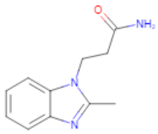<br>Z53116498	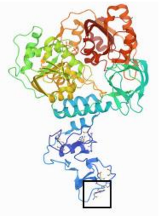<br>Zinc B	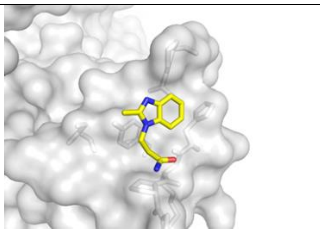<br>Zinc B	2.43
5RLM	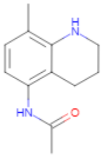<br>Z1650168321	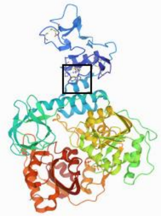<br>Zinc B	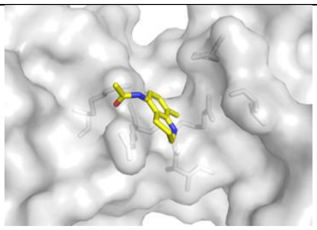<br>Zinc B	1.86
5RMI	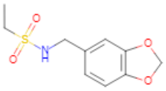<br>Z53860899	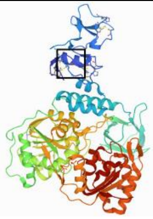<br>Zinc B	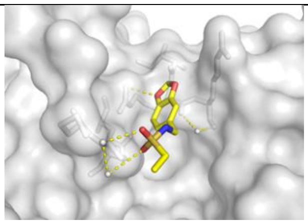<br>Zinc B	2.12
5RLF	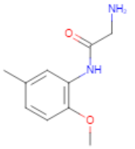<br>Z235341991	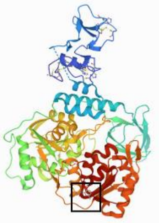<br>2A domain A	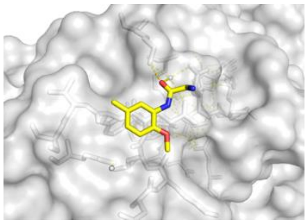<br>2A domain A	2.23
5RLQ	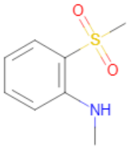<br>Z285782452	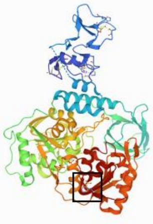<br>2A domain A	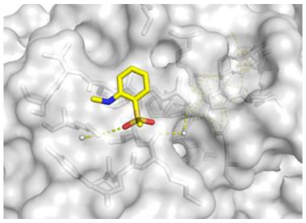<br>2A domain A	2.23

5RLG	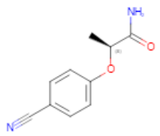<br>Z19739650	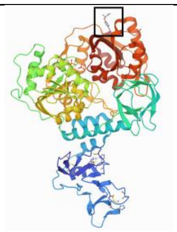<br>2A domain B	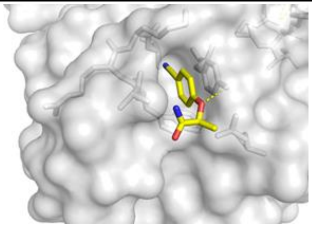<br>2A domain B	1.96
5RM3	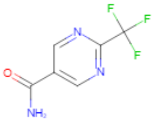<br>Z1745658474	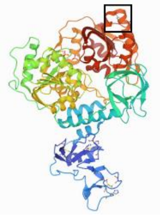<br>C-terminus B	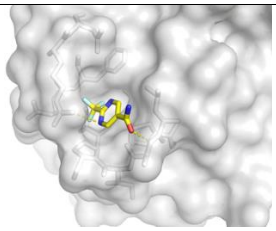<br>C-terminus B	2.09
5RM6	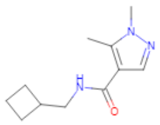<br>Z396380540	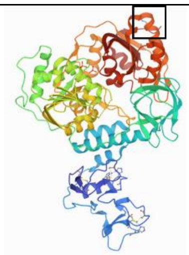<br>C-terminus B	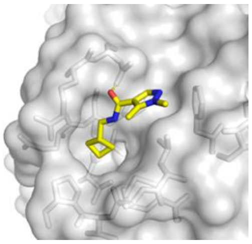<br>C-terminus B	2.13
5RM9	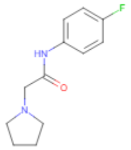<br>Z2856434942	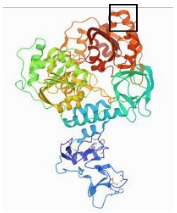<br>C-terminus B	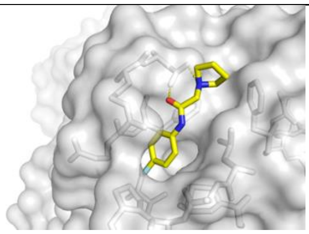<br>C-terminus B	2.08
5RMG	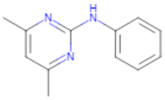<br>Z285675722	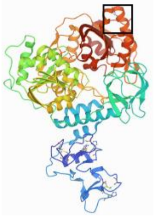<br>C-terminus B	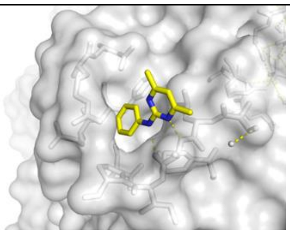<br>C-terminus B	2.12
5RMJ	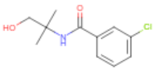<br>Z68299550	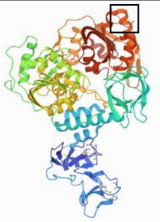<br>C-terminus B	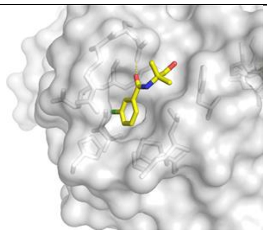<br>C-terminus B	2.10

5RMB	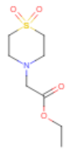 Z2856434920	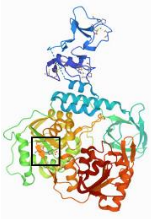 Interface 1A-2A A	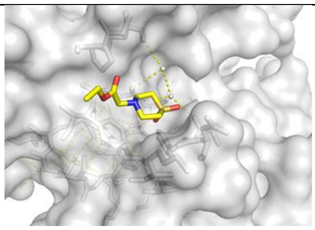 Interface 1A-2A A	2.21
5RMF	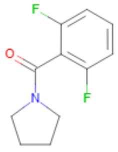 Z54226006	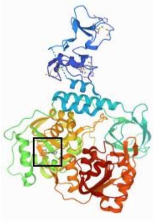 Interface 1A-2A A	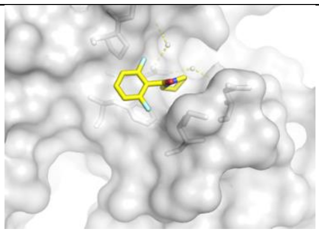 Interface 1A-2A A	2.23
5RM4	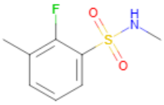 Z1639162606	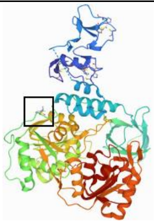 1A domain A	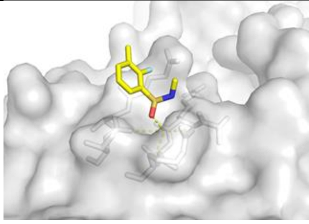 1A domain A	2.96
5RM5	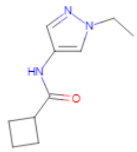 Z373768900	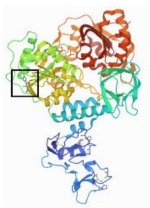 1A domain B	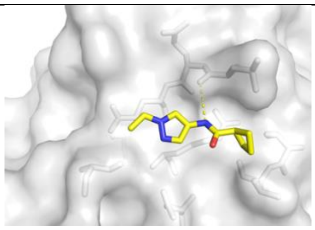 1A domain B	2.06
5RM8	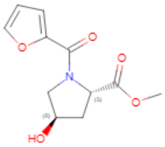 Z1614545742	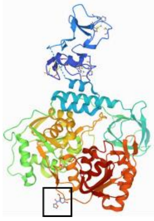 2A domain A	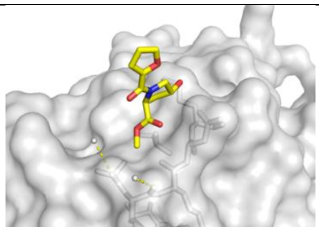 2A domain A	2.14
5RMA	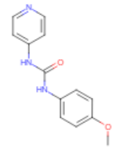 Z321318226	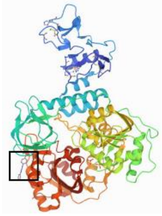 1B-2A interface B	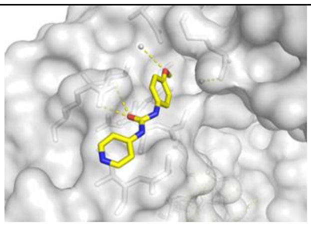 1B-2A interface B	1.89

5RMH	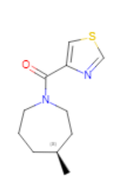 Z1101755952	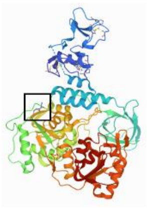 1A domain A	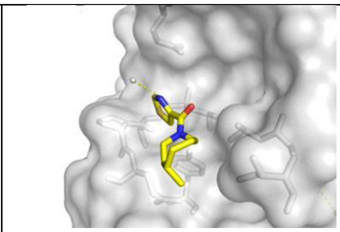 1A domain A	2.02

Supplementary Table 2 – Structures, accession codes, resolutions and binding sites of NSP13 fragment hits.

PDB ID	PANDDA event map	$2F_o$-$1F_c$ Map (1σ)	$2F_o$-$1F_c$ Omit Map (1σ)
5RL6	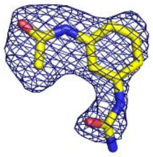	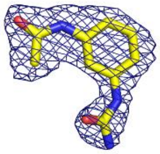	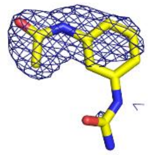
5RL7(1)	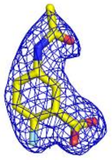	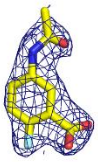	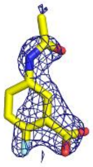
5RL7(2)	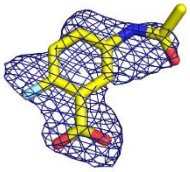	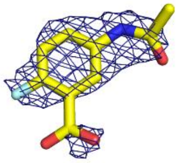	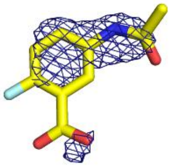
5RL8	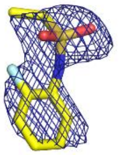	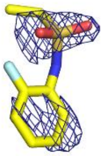	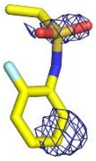
5RL9	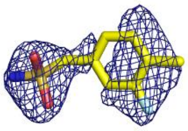	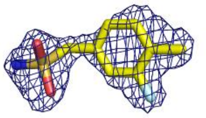	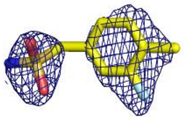

5RLB	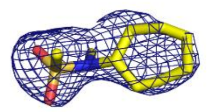	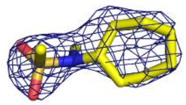	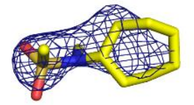
5RLC	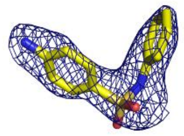	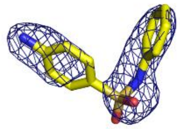	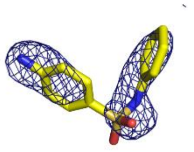
5RLD	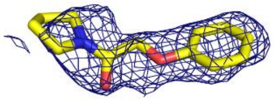	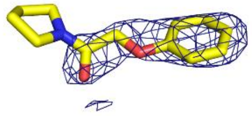	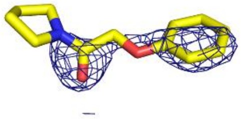
5RLE	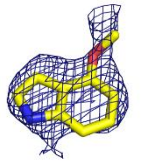	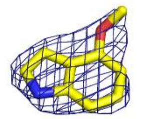	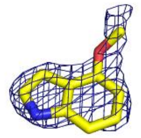
5RLF	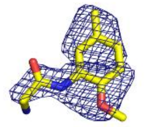	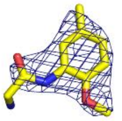	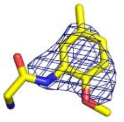
5RLG	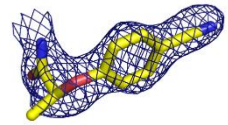	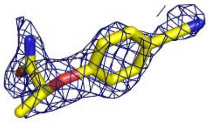	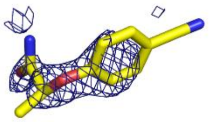
5RLH	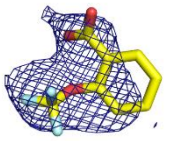	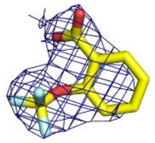	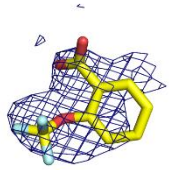

5RLI (1)	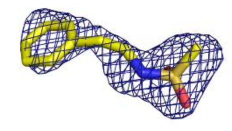	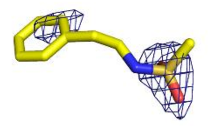	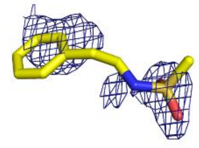
5RLI (2)	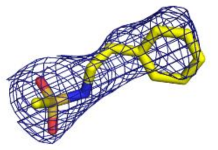	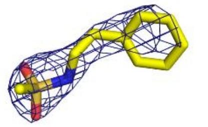	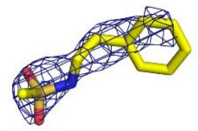
5RLI (3)	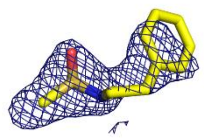	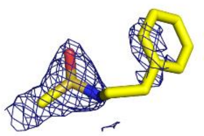	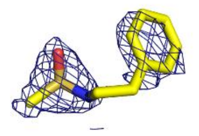
5RLJ	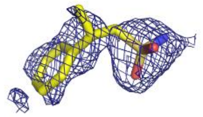	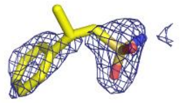	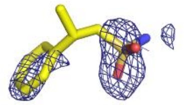
5RLK	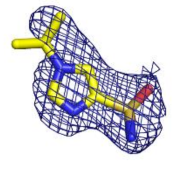	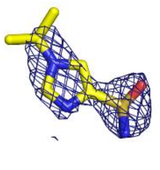	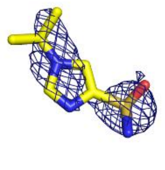
5RLL	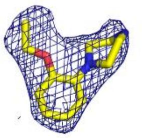	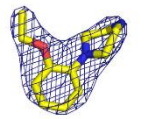	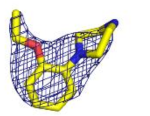
5RLM	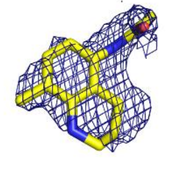	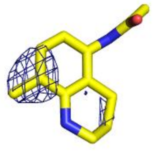	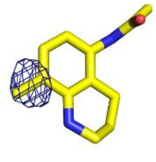

5RLN	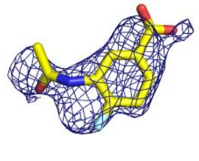	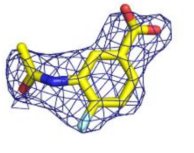	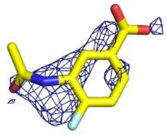
5RLO	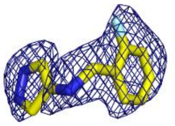	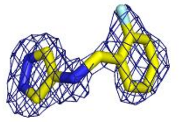	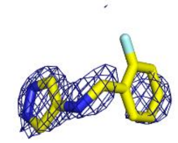
5RLP	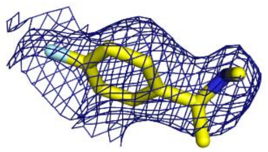	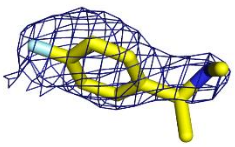	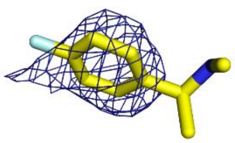
5RLQ	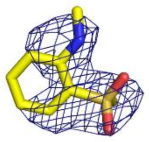	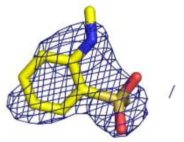	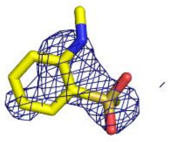
5RLR	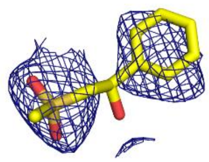	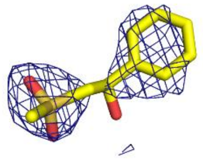	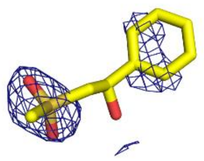
5RLS	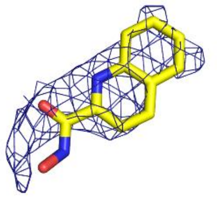	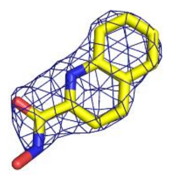	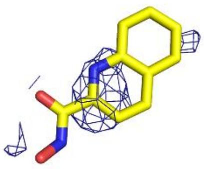
5RLT	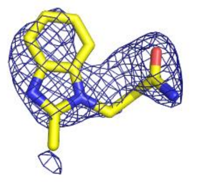	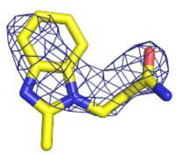	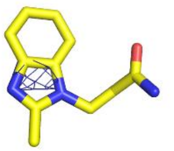

5RLU(1)	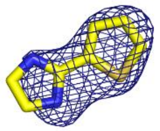	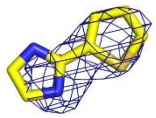	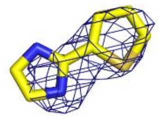
5RLU(2)	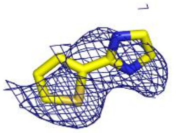	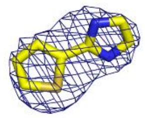	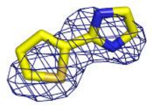
5RLV(1)	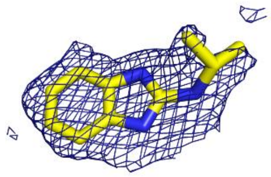	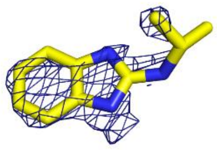	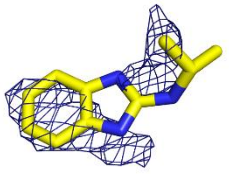
5RLV(2)	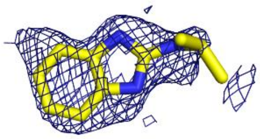	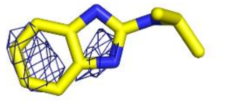	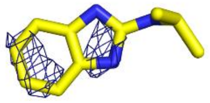
5RLV(3)	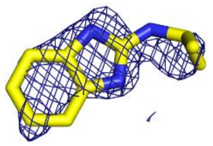	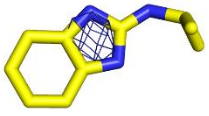	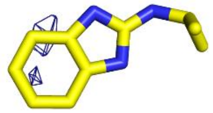
5RLW(1)	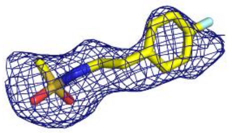	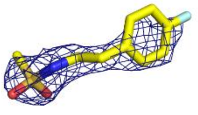	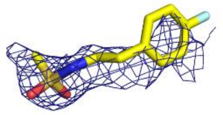
5RLW(2)	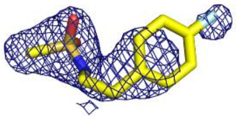	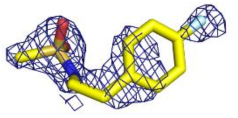	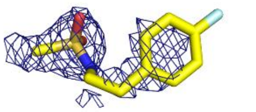

5RLY(1)	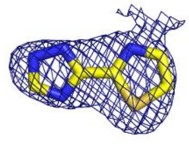	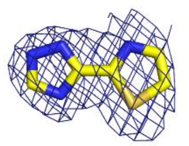	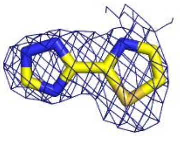
5RLY(2)	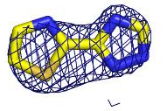	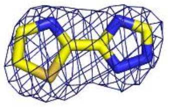	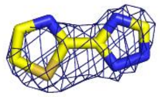
5RLY(3)	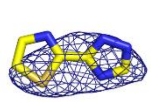	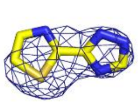	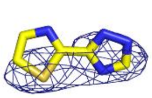
5RLZ	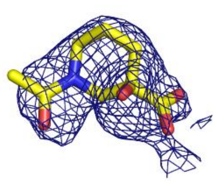	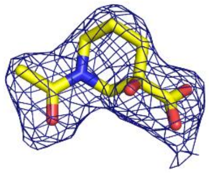	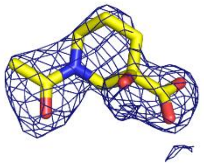
5RM0	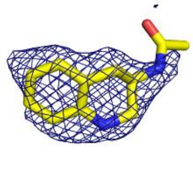	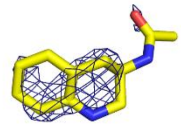	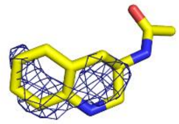
5RM1	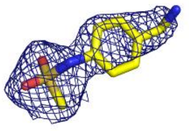	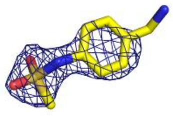	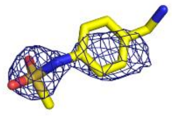
5RM2(1)	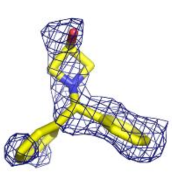	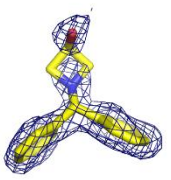	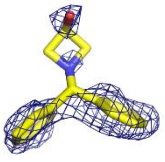

5RM2(2)	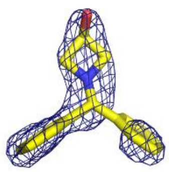	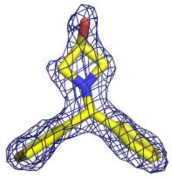	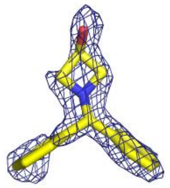
5RM3	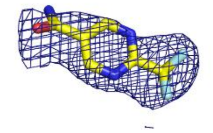	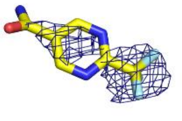	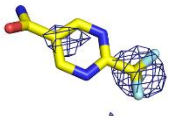
5RM4	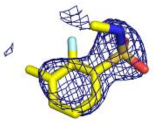	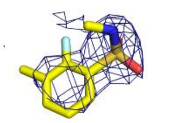	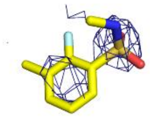
5RM5	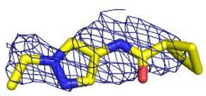	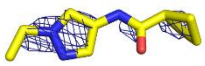	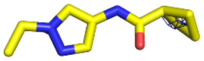
5RM6	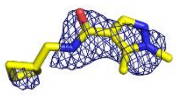	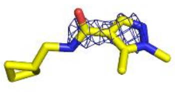	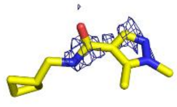
5RM7	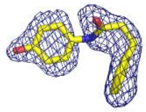	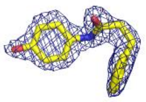	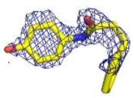
5RM8	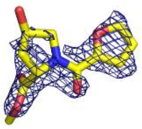	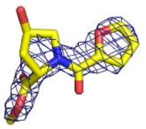	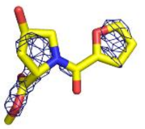

5RM9	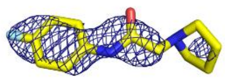	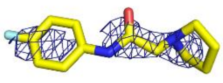	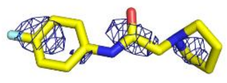
5RMA	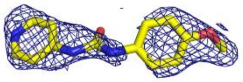	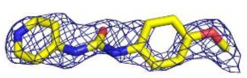	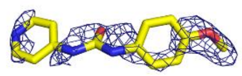
5RMB	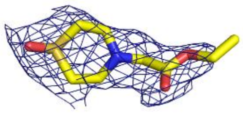	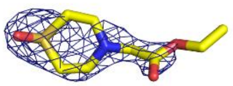	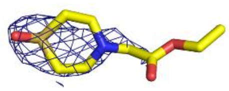
5RMC	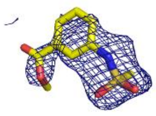	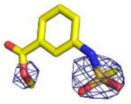	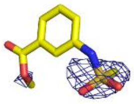
5RMD(1)	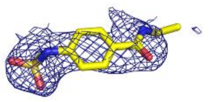	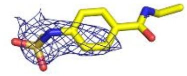	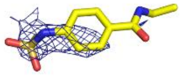
5RMD(2)	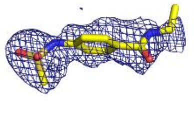	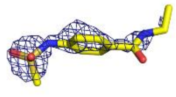	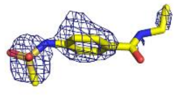
5RME	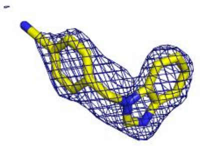	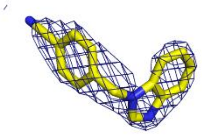	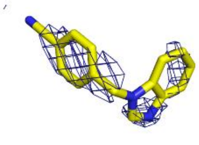

5RMF	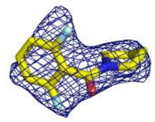	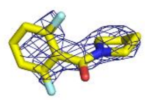	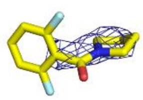
5RMG	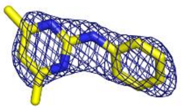	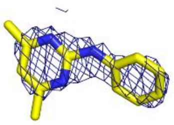	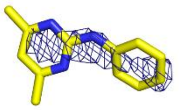
5RMH	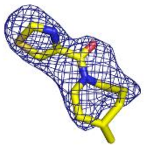	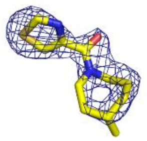	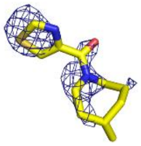
5RMI	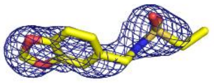	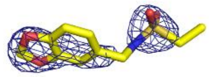	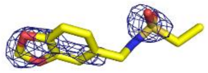
5RMJ	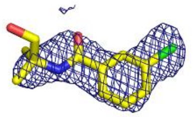	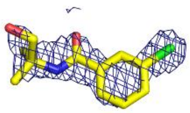	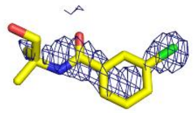
5RMK	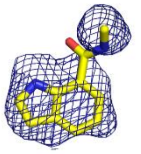	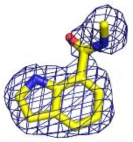	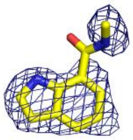
5RML	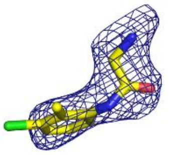	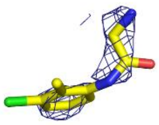	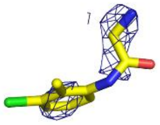

5RMM	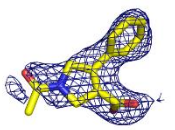	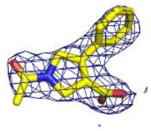	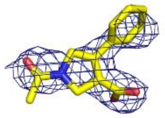

Supplementary Table 3 - Electron density maps of the NSP13 fragment hits. PANDDA event maps are shown in the left-hand panel and are contoured individually. The center and right-hand panels show sigma-A weighted maps calculated with (center) and without (right) the ligand coordinates and are contoured at 1 σ throughout to enable comparison of electron density levels between fragment hits.

NSP13 DNA	ATGCACCATCATCATCATCATTCTTCTGGTGTGGATAACAAGTTCAACAAGGAGCGTCGAAGAGCTCGCCGTGAAATT<br>CGCCATCTGCCGAACCTGAACCGCGAACAGCGTCGCGCATTTATTCGCAGCCTGCGCGATGATCCGAGCCAGAGCG<br>CGAACCTGCTGGCGGAAGCGAAGAAGCTGAACGATGCGCAGCCGAAGGGTACCGAGAACCTGTACTTCCAATCCAT<br>GGCTGTTGGTGCATGCGTTTTGTGTAATAGTCAAACTTCTTTGCGTTGCGGCGCCTGCATCCGCCGCCCATTTCTGTG<br>CTGTAAATGTTGCTACGATCATGTGATTAGCACGTCGCATAAGCTGGTGCTGTCGGTCAACCCGTATGTTTGTAATGC<br>GCCCGGTTGTGACGTCACGGATGTTACCCAGCTCTATCTGGGTGGTATGTCTTACTACTGCAAGTCACATAAGCCGCC<br>GATTTCATTTCCTCTCTGCGCGAATGGCCAGGTGTTTGGTTTGTATAAAAACACCTGCGTGGGGTCTGATAATGTAAC<br>CGATTTTAACGCGATTGCTACGTGTGACTGGACCAACGCCGGCGATTATATCCTTGCCAACACCTGCACTGAACGTCT<br>TAAACTCTTCGCGGCCGAAACCCTGAAAGCAACCGAAGAAACGTTCAAACTGAGCTATGGTATCGCGACAGTACGCG<br>AAGTACTCAGTGACCGGGAGTTGCACCTGTCGTGGGAAGTCGGTAAACCTCGCCCCCCGTTAAACCGCAACTACGTG<br>TTCACCGGCTATCGCGTTACCAAAAACAGCAAGGTGCAAATTGGTGAGTACACCTTTGAGAAAGGCGACTATGGTGA<br>TGCCGTAGTATACCGCGGCACCACGACTTACAAACTGAATGTGGGTGATTATTTTGTATTAACCTCACATACCGTAAT<br>GCCGTTGAGCGCCCCCACCCTCGTACCGCAGGAACACTATGTACGTATCACGGGTTTATACCCGACGCTGAACATCA<br>GCGATGAGTTTAGCAGTAACGTTGCTAACTACCAGAAAGTGGGTATGCAAAAATACTCGACGTTACAGGGCCCGCCA<br>GGTACTGGAAAGTCTCATTTCGCCATCGGCTTAGCCTTATACTATCCATCAGCTCGCATTGTTTACACGGCCTGCTCTC<br>ATGCTGCAGTGGACGCCTTATGCGAGAAGGCGTTAAAATATCTGCCGATTGATAAATGCTCCCGTATTATCCCGGCG<br>CGGGCGCGGGTCGAGTGTTTTGACAAATTTAAAGTGAACAGCACACTGGAACAGTATGTTTTTTGTACGGTGAATGC<br>TCTCCCAGAGACAACTGCCGATATCGTCGTGTTCGACGAAATCAGCATGGCCACTAACTACGATCTGTCGGTCGTTAA<br>CGCGCGTCTCCGCGCTAAGCATTATGTTTACATTGGCGATCCGGCCCAGCTGCCGGCTCCACGCACGCTCCTGACTA<br>AAGGTACTCTGGAACCGGAATATTTTAATAGCGTATGCCGTCTGATGAAAACGATTGGACCTGATATGTTCCTGGGA<br>ACTTGCCGCCGGTGTCCGGCCGAGATCGTTGATACGGTTTCTGCTCTGGTTTATGACAATAAATTAAAAGCTCACAAA<br>GATAAAAGCGCGCAATGCTTCAAAATGTTTTATAAAGGTGTAATTACCCACGATGTGAGTAGCGCGATTAACCGCCC<br>GCAAATTGGCGTTGTCCGTGAATTCCTGACCCGGAATCCTGCGTGGCGCAAAGCAGTGTTCATTAGCCCATATAATA<br>GTCAAAACGCGGTAGCGTCCAAGATTTTGGGTCTGCCGACACAAACCGTGGACTCCAGCCAGGGCAGTGAGTACGA<br>CTATGTGATTTTTACCCAGACCACTGAGACAGCACACTCCTGCAACGTGAACCGCTTCAATGTAGCGATCACCCGTGC<br>GAAGGTAGGTATTTTGTGCATCATGTCCGACCGTGATCTGTATGACAAATTGCAGTTTACCAGCCTGGAAATCCCTCG<br>CCGTAACGTGGCAACCCTTCAATAACAGTAAAGGTGGATACGGATCCGAATTCGAGCTCCGTCGACAAGCTTGCGGC<br>CGCACTCGAGCACCACCACCACCACCACTGA

NSP13 Protein	**MHHHHHHSSGVDNKFNKERRRARREIRHLPNLNREQRRAFIRSLRDDPSQSANLLAEAKKLNDAQPKGTENLYFQS**MAVGACVLCNSQTSLRCGACIRRPFLCCKCCYDHVISTSHKLVLSVNPYVCNAPGCDVTDVTQLYLGGMSYYCKSHKPPISFPLCANGQVFGLYKNTCVGSDNVTDFNAIATCDWTNAGDYILANTCTERLKLFAAETLKATEETFKLSYGIATVREVLSDRELHLSWEVGKPRPPLNRNYVFTGYRVTKNSKVQIGEYTFEKGDYGDAVVYRGTTTYKLNVGDYFVLTSHTVMPLSAPTLVPQEHYVRITGLYPTLNISDEFSSNVANYQKVGMQKYSTLQGPPGTGKSHFAIGLALYYPSARIVYTACSHAAVDALCEKALKYLPIDKCSRIIPARARVECFDKFKVNSTLEQYVFCTVNALPETTADIVVFDEISMATNYDLSVVNARLRAKHYVYIGDPAQLPAPRTLLTKGTLEPEYFNSVCRLMKTIGPDMFLGTCRRCPAEIVDTVSALVYDNKLKAHKDKSAQCFKMFYKGVITHDVSSAINRPQIGVVREFLTRNPAWRKAVFISPYNSQNAVASKILGLPTQTVDSSQGSEYDYVIFTQTTETAHSCNVNRFNVAITRAKVGILCIMSDRDLYDKLQFTSLEIPRRNVATLQ

Supplementary Table 4 – DNA and protein sequences for the codon optimized NSP13 construct used in this study. The bold sequence is removed in purification by TEV protease cleavage.
