# Supplementary material for: Structure, mechanism and crystallographic fragment screening of the SARS-CoV-2 NSP13 helicase
Source: Nat Commun. 2021 Aug 11;12:4848. doi: 10.1038/s41467-021-25166-6 (PMC8358061; doi:10.1038/s41467-021-25166-6)
Supplement: Supplementary file 3 — Description of Additional Supplementary Files [file 41467_2021_25166_MOESM3_ESM.pdf]

Description of Additional Supplementary Files

File Name: Supplementary Data 1
Description: Excel file showing a table with crystallographic data collection and refinement statistics for all fragment datasets obtained in this study.

File Name: Supplementary Movie 1
Description: Animation showing the proposed NSP13 catalytic cycle. The animation starts in the APO open form, ATP binding induces a conformational change to the closed form with the 1A domain gripping the RNA and the 2A domain sliding. ATP hydrolysis triggers another conformational change back to the open form with the 2A domain gripping the RNA and 1A domain sliding. ADP is released before phosphate.
